# DeSmoke-LAP: improved unpaired image-to-image translation for desmoking in laparoscopic surgery

**DOI:** 10.1007/s11548-022-02595-2

**Published:** 2022-03-30

**Authors:** Yirou Pan, Sophia Bano, Francisco Vasconcelos, Hyun Park, Taikyeong Ted. Jeong, Danail Stoyanov

**Affiliations:** 1 Wellcome/EPSRC Centre for Interventional and Surgical Sciences, Department of Computer Science, University College London, London, UK; 2 Department of Obstetrics and Gynecology, CHA Bundang Medical Center, CHA University, Seongnam, South Korea; 3 School of Artificial Intelligence Convergence, Hallym University, Chuncheon, South Korea

**Keywords:** Desmoking, Robotic-assisted laparoscopic hysterectomy, Deep learning, Generative adversarial network

## Abstract

**Purpose:**

Robotic-assisted laparoscopic surgery has become the trend in medicine thanks to its convenience and lower risk of infection against traditional open surgery. However, the visibility during these procedures may severely deteriorate due to electrocauterisation which generates smoke in the operating cavity. This decreased visibility hinders the procedural time and surgical performance. Recent deep learning-based techniques have shown the potential for smoke and glare removal, but few targets laparoscopic videos.

**Method:**

We propose DeSmoke-LAP, a new method for removing smoke from real robotic laparoscopic hysterectomy videos. The proposed method is based on the unpaired image-to-image cycle-consistent generative adversarial network in which two novel loss functions, namely, inter-channel discrepancies and dark channel prior, are integrated to facilitate smoke removal while maintaining the true semantics and illumination of the scene.

**Results:**

DeSmoke-LAP is compared with several state-of-the-art desmoking methods qualitatively and quantitatively using referenceless image quality metrics on 10 laparoscopic hysterectomy videos through 5-fold cross-validation.

**Conclusion:**

DeSmoke-LAP outperformed existing methods and generated smoke-free images without applying ground truths (paired images) and atmospheric scattering model. This shows distinctive achievement in dehazing in surgery, even in scenarios with partial inhomogenenous smoke. Our code and hysterectomy dataset will be made publicly available at https://www.ucl.ac.uk/interventional-surgical-sciences/weiss-open-research/weiss-open-data-server/desmoke-lap.

## Introduction

**Fig. 1:**
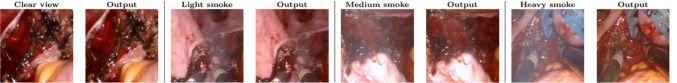
Sample laparoscopic frames showing different grades of smoke in input and desmoked output from the proposed DeSmoke-LAP method

In laparoscopic surgery, the risks of bleeding can be reduced using instruments with electrocauterisation capabilities, in which a heating source is directly applied to tissue for dissection. Such electric instruments have been adapted to robotic-assisted surgery platforms such as the da Vinci Xi in the context of e.g. performing cholecystectomy and hysterectomy. One of the challenges in electrocauterisation is the production of smoke that hinders the visibility of the operative site through the laparoscopic camera. This may require the surgeon to stop any action until visibility is re-established. As demonstrated in [1], this leads to an increase in the operation time as well as surgeon’s anxiety. Figure 1 shows sample laparoscopic images with clear view (no smoke) and light, medium and high density of smoke.

A substantial number of computer vision techniques have been proposed before to restore visibility in hazy images. These include traditional computer vision methods, generative adversarial networks (GANs) for paired image-to-image translation and cycle-consistent generative adversarial networks (CycleGANs) for unpaired image-to-image translation. Traditional methods use neural networks [2] or variational interference [3, 4] for image desmoking, whose generator is simply updated according to the provided database. On the other hand, GAN model updates the generator by the backpropagation from discriminator, which helps in obtaining more reliable results. Paired image-to-image translation GANs [5] require the same images with and without ground-truth hazy conditions during training and thus rely on synthetic training data. In contrast, GANs for unpaired data [6, 7] can be trained simply on arbitrary examples of clear and hazy images, without the need of ground truths or generative physical models, thus offering more flexibility in terms of training data.

Several physical models, including atmospheric scattering model and dark channel prior, have been utilised to model smoke parameters efficiently [2, 5, 8, 9]. The purpose of the atmospheric scattering model is to simulate the smoke component by relating global atmospheric light to the transmission map [2, 8]. However, generated smoke cannot be distributed uniformly and thus cannot be simply computed by the scattering model. The dark channel prior is shown to model haze [5], but no attempts have been done to investigate its use within a loss function to train a dehazing model using unpaired real data. Due to the difficulty in obtaining paired images for real data, quantitative evaluation of these methods mostly relies on synthetic data.

In this paper, we propose DeSmoke-LAP, a dehazing technique to improve the visibility of laparoscopic scenes during electrocauterisation. We use an unpaired surgical image dehazing network which is based on CycleGAN [6]. Our proposed method enhances the CycleGAN network by introducing the inter-channel discrepancies and the dark channel prior as part of the loss function during network training. These losses help in modelling different smoke components and lead to smoke-free images with visually higher quality. We created a dataset of clear view and hazy images from 10 laparoscopic hysterectomy videos and use cross-validation for evaluation. Additionally, we perform validation on continuous clips, containing varying smoke density, from each video to assess real operation scenarios. Since paired ground truths are not available, we propose to utilise three existing referenceless metrics for the performance evaluation. Through both quantitative and qualitative comparative analysis with the existing methods, we show that our proposed method achieves better performance. The main contributions of this paper are as follows:

- We develop enhanced CycleGAN which focuses on smoke removal in laparoscopic surgery using unpaired image-to-image translation, without utilising atmospheric scattering models or ground truths during model training.
- We introduce additional loss functions on inter-channel discrepancies and dark channel prior that allows qualifying remaining smoke component in the generated image, aiding cycle-consistency loss and adversarial loss.
- We introduce the use of referenceless image quality metrics for evaluation which are designed to measure image quality in the absence of ground truth.
- The utilised dataset that includes 6000 clear and hazy images extracted from 10 laparoscopic videos and 10 video clips are made publicly available^1^, providing a benchmark for unpaired laparoscopic image desmoking.

## Proposed method

**Fig. 2:**
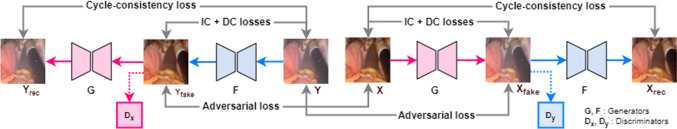
An overview of the proposed DeSmoke-LAP method. The inter-channel (IC) discrepancies and dark channel (DC) prior are introduced to qualify the remaining smoke, aiding cycle-consistency and adversarial losses in smoke removal

The proposed DeSmoke-LAP model is designed for unpaired image-to-image translation in two domains based on the architecture of CycleGAN, where two additional loss functions are designed for inter-channel differences and dark channel prior (as shown in Fig. 2). These loss functions, added for discriminating, aim to capture the remaining smoke covered on the generated image and promote the optimisation of the generator in the next iteration.

### Cycle-consistent generative adversarial network

CycleGAN architecture forms the backbone of our proposed method, which is an improved GAN [10] that uses adversarial and cycle-consistency losses for unpaired image-to-image translation from source *X* to target *Y* domains. GAN is composed of generator and discriminator, where the purpose of the generator is to synthesise examples realistic enough to fool the discriminator, while the discriminator aims to correctly distinguish between real and synthetic images. The weights of these two models are updated dynamically to achieve a stabilised balance. Given unpaired clear (smoke-free) images $\{x_i\}_{i=1}^{N}$ where $x_i \in X$ and hazy images $\{y_j\}_{j=1}^{M}$ where $y_j \in Y$, the goal is to learn the mapping between *X* and *Y*. Two discriminators are implemented in the network, where $D_X$ is applied to distinguish between clear images *X* and translated data from hazy images *F*(*Y*) and $D_Y$ distinguishes hazy images *Y* and translated data from clear image *F*(*X*) (Fig. 2). The adversarial loss measures the deviation between the translated image from one domain and the real sample in the other domain. It is applied to both generator and discriminator, where discriminator aims to maximise the loss and generator aims to minimise it.

1 $$\begin{aligned}&\mathcal {L}(G, D_Y, X, Y) = \mathbb {E}_{y\sim p_{data}(y)}[\log D_Y(y)]\nonumber \\&\quad + \mathbb {E}_{x\sim p_{data}(x)}[\log (1-D_Y(G(x)))], \end{aligned}$$

2 $$\begin{aligned}&\mathcal {L}(F, D_X, X, Y) = \mathbb {E}_{x\sim p_{data}(x)}[\log D_X(x)] \nonumber \\&\quad + \mathbb {E}_{y\sim p_{data}(y)}[\log (1-D_X(F(Y)))], \end{aligned}$$

where $x\sim p_{data}(x)$ and $y\sim p_{data}(y)$ denote the data distributions of the two domains. The objective of generative adversarial loss is summarised as:

3 $$\begin{aligned}&\mathcal {L}_{GAN}(G,F,D_X,D_Y) = \min _{G} \max _{D_Y} \mathcal {L}(G, D_Y, X, Y) \nonumber \\&\quad + \min _{F} \max _{D_X} \mathcal {L}(F, D_X, Y, X). \end{aligned}$$

The cycle-consistency loss is evaluated to improve the functionality of generators, which aims to assess the difference between the real data in one domain and data that translated forward and back to the origin domain. It judges the recovery with two translations, forward cycle consistency: $x \rightarrow G(x) \rightarrow F(G(x)) \approx x$ and backward cycle consistency: $y \rightarrow F(y) \rightarrow G(F(y)) \approx y$.

4 $$\begin{aligned} \mathcal {L}_{cyc}(G, F)= & {} \mathbb {E}_{x\sim p_{data}(x)} [\Vert x - F(G(x))\Vert _1] \nonumber \\&+ \mathbb {E}_{y\sim p_{data}(y)} [\Vert y - G(F(y))\Vert _1] \end{aligned}$$

### DeSmoke-LAP: desmoking in laparoscopic surgery

CycleGAN alone cannot eliminate smoke from laparoscopic video frames since it does not learn to optimise the model using priors specific to the smoke. Therefore, we propose DeSmoke-LAP, a desmoking approach for laparoscopic surgery that targets hazy-to-clear translation by introducing two additional loss functions, namely, inter-channel loss and dark channel loss, to the discriminator of each domain. These losses allow measuring the remaining smoke components in the generated image by evaluating the differences between images before and after processing them through the generator.

#### Inter-channel (IC) Loss

Inter-channel discrepancies [11, 12] describe the difference between any two channels of a pixel in the image by the use of absolute norm,

5 $$\begin{aligned} \Psi (P) = \Vert P_R - P_G \Vert _1 + \Vert P_G - P_B \Vert _1 + \Vert P_B - P_R \Vert _1, \end{aligned}$$

where *P* denotes a pixel in the image, and $P_R$, $P_G$ and $P_D$, respectively, represent the R, G, D channel of the pixel. The value of channels in the pixel is normalised between 0 and 1. Thus, the loss of an image can be measured by the mean value of norms for all pixels in that image, where *x* corresponds to the selected image and *n* represents the total amount of pixels, $P_{i...n} \in X$.

6 $$\begin{aligned} I^{IC}(X) = \frac{1}{n} \sum _{i=1}^{n} \Psi (P_i), \end{aligned}$$

According to the observations of He et al. [9], the inter-channel difference of a pixel in equation 6 relates to the level of blur and a small value is obtained when there is heavy smoke covered on that pixel. The normalised difference will be reflected in the discriminator and contribute to the development of the generator. Based on the analysis on smoke in image, a low value indicates a high level of smoke for a pixel. Thus, we consider that use 1 as the boundary in the calculation to ensure that the function results in large impacts to the generator if the divergence is small. Our network works between the clear and hazy domains, when performing hazy-to-clear translation, it is intended to generate a fake image with less smoke, and hence, the loss detects the hazy components. If the target of the translation is a hazy image, the corresponding discriminator is developed by the smoke-free sections. The inter-channel loss used in the network is given by,

7 $$\begin{aligned} \mathcal {L}_{IC} = f(I^{IC}(G(x))) + f(I^{IC}(F(y))), \end{aligned}$$

where

8 $$\begin{aligned} f(x) = \left\{ \begin{array}{lr} 1 - \Vert x - 1 \Vert , &{} clean \rightarrow hazy \\ \Vert x - 1 \Vert , &{} hazy \rightarrow clean. \end{array} \right. \end{aligned}$$

#### Dark channel (DC) loss

Inspired by [5, 9, 13], we assess hazy components in the image using the dark channel prior, which measures the intensity of the image and reveals its luminous density. This is defined as the minimum value in the RGB channels

9 $$\begin{aligned} I^{dark}(x) = \min _{y \in \Omega (x)} ( \min _{c \in {r,g,b} } I^c (y)), \end{aligned}$$

where $I^c$ is a colour channel of the arbitrary image *I* and $\Omega (x)$ is a local patch centred at *x*. If the image is smoke-free, $I^{dark} \rightarrow 0$. We observe that most of the pixels in a clean laparoscopic image have a low-density value, but few smoke-free pixels still output high value due to the luminance effect and light reflection. To fairly and reasonably access the dark channel of the sample input, the dark channel loss of an image is measured as the average value by looping through all pixels, filtering out extra-high or extra-low value. Adding the refinement stage for the dark channel by applying the soft matting algorithm [9] helps to measure and highlight the edge and profile of objects, maintaining more details in the image, and the algorithm is executed in our model as well. The DC loss is, respectively, added to the two discriminators, and if most of the sample is covered by smoke, the loss will be large, promoting the parameter optimisation of the generator.

10 $$\begin{aligned} \mathcal {L}_{DC} = \overline{I_{dark}(F(y))} + \overline{I_{dark}(G(x))} \end{aligned}$$

#### Combined loss function

The full objective of loss function for the proposed Desmoke-LAP is given by

11 $$\begin{aligned}&\mathcal {L}(G,F,D_x,D_y) = \mathcal {L}_{CycleGAN}(G,F,D_x,D_y) \nonumber \\&\quad + \alpha \cdot \mathcal {L}_{IC}(D_x,D_y) + \beta \cdot \mathcal {L}_{DC}(D_x, D_y), \end{aligned}$$

12 $$\begin{aligned}&\mathcal {L}_{CycleGAN}(G,F,D_x,D_y) = \mathcal {L}_{GAN}(G,F,D_X,D_Y)\nonumber \\&\quad + \mathcal {L}_{cyc}(G, F). \end{aligned}$$

To regulate the DC loss, $\beta$ is added in Eq. 11 where its value is selected through experimentation (refer to Sect. 4.2), and $\alpha$ is used to maintain the balance between IC and cycle loss. *G* and *F*, respectively, stand for generators for domain *X* and *Y*, and $D_x$ and $D_y$ are two discriminators in two domains.

## Referenceless evaluation metrics

Since all our data are unpaired and collected from real robotic surgery, ground-truth (paired clear and hazy) images are not available. Therefore, metrics commonly used in haze and smoke removal evaluation such as mean squared error (MSE), peak signal-to-noise ratio (PSNR), structural similarity index measure (SSIM), etc., are not applicable. We rely on several referenceless image quality metrics for evaluating the performance of the resulting desmoke images, but these metrics are designed using real-world images having different distribution than surgical images and cannot be solely considered as a performance evaluation criterion. Three referenceless metrics evaluate reconstructed images based on the fog density, image blurriness and edge restoration, and they are briefly explained below:

**Fog aware density evaluator (FADE)** [14] is used to compute the fog density of the image, where a higher FADE value means there is more fog covered on the image. It is constructed in accordance with natural scene statistics (NSS) and fog aware statistical features.

**Just noticeable blur metric (JNBM)** [15] measures the perceptual sharpness of the image, where a lower value results from low sharpness. It focuses on the behaviour of the human visual system to sharpness at different contrast levels and accesses the blurriness of edges in the image.

**Restoring edge assessment (REA)** [16] assesses the edge restoration of the image, which differentiates between the original and the reconstructed image. A higher value of REA indicates better restoration of the edges.

## Dataset and experimental setup

### Data organisation

We collected 10 robot-assisted laparoscopic hysterectomy procedure recordings. Active instrument labels at the bottom of the video-feed assisted in manually annotating hazy and clear images. These videos were decomposed into frames at 1 fps. 300 clear and 300 hazy images per video were selected to form our dataset. In total, 3000 clear and 3000 hazy images were selected from 10 sampled videos, where the images were cropped to remove video display and resized to $720 \times 540$ pixel resolution while maintaining the aspect ratio. The organised data contain both inter-patient and intra-patient variabilities in the scene, adding diversity to the dataset. Intra-patient variability is experienced due to the movement of the camera in the surgical operating field. The collected images contain various levels of haze that were split into light, medium, heavy and partial smoke. Moreover, a sequence of 50 frames is selected as a short clip from each video, such that these clips also capture frames with motion blur. These clips are used to analyse consistency of desmoking algorithms across frames. The dataset summary is provided in the supplementary Sec. 1.

### Training details

**Fig. 3:**
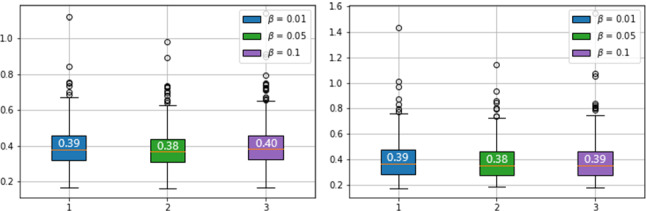
FADE value of clear (left) and hazy (right) images in fold 3 with various $\beta$

**Fig. 4:**
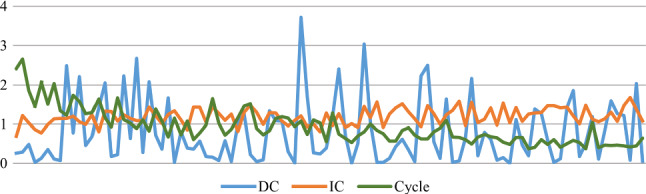
Log of cycle loss, DC loss and IC loss in 100 epoches

For all experiments, models are trained using an Nvida 16GB V100 GPU and batch size of 4. The DeSmoke-LAP utilises a ResNet generator and a PatchGAN discriminator along with a least-squares GANs objective for each network, following the implementation by Zhu et al. [6]. The learning rate is set to 0.002 for the first 50 iterations and linearly decays to 0 in the latter 50 epochs. To test and verify the superiority of the combined losses, the model is trained with DC and IC losses independently. To control the effect of the dark channel prior efficiently, testing was completed on one fold with various values of $\beta$. The model trained with a larger $\beta$ gave a lower FADE value indicated in Fig. 3. The median FADE value is lowest at $\beta =0.05$, leading to outputs with less smoke, and thus, $\beta$ in equation 11 is set to 0.05. To maintain the balance among cycle loss, IC loss and DC loss, the IC loss is normalised in the calculation and the weight is set at 3 to ensure that cycle and IC loss lie in the same range, while DC loss stays at higher value resulting in a larger impact, shown in Fig. 4.

To investigate the performance of our proposed model, five-fold cross-validation is used, with each fold containing image samples from 2 videos. A sequence of 50 continuous frames from each test video aids to evaluate the network. The data are cropped at random positions to $256 \times 256$ resolution for data augmentation, creating more patches before training.

We perform quantitative and qualitative comparisons, along with a qualitative user study, of Desmoke-LAP with CycleGAN [6], FastCUT [17], Cycle-Dehaze [7] and Colores et al. [5] methods. FastCUT [17] improves over CycleGAN by providing a faster training network for image translation, utilising the advantages of contrastive learning. Cycle-Dehaze [7] is an enhanced CycleGAN for image dehazing that employs cyclic perceptual-consistency loss to maintain the original structure of the image. Colores et al. [5] fused the dark channel prior with inputs before passing it to the generator for learning paired image-to-image translation. Experiments were performed with hazy images synthesised by adding smoke to the input image. Since our data is unpaired, retraining of this model is not possible. Therefore, we use the pre-trained Colores et al. [5] model on our dataset.

The training and testing times have also been recorded for further investigation (attached as Table 12 in supplementary material). It takes more than 9 hours to train CycleGAN, FastCut and the proposed method.

## Results and discussion

Quantitative comparison of proposed DeSmoke-LAP (IC$+$DC) and the existing models using five-fold cross-validation with average FADE, JNBM and REA metrics and their standard deviation over all folds is presented in Table 1. The results over each fold are provided in the supplementary material Sec. 2. It reveals that the model with both loss functions outperformed on all metrics, whereas the performance of the model with only one loss was attenuated on either haze or contrast levels. Focusing on quantitative results, DeSmoke-LAP performance was marginally lower than Colores et al. [5] and Cycle-Dehaze [7], though DeSmoke-LAP outperformed other traditional unpaired translation methods [6, 17]. However, when visually analysing the methods under comparison (Fig. 1–2 in Supplementary), we observe that though JNBM value of Colores et al. and Cycle-Dehaze and DeSmoke-LAP are comparable, DeSmoke-LAP removed smoke while retaining scene semantic, i.e. without overexposing and attenuating the image intensity. Besides, we observe that all referenceless metrics follow the same trend.

**Table 1:** Quantitative comparison through five-fold cross-validation on the organised clear and hazy images dataset. Mean and standard deviation of the 3 metrics are reported. Lower FADE, and higher JNBM and REA values are better

	FADE		JNBM		REA	
	Clear	Hazy	Clear	Hazy	Clear	Hazy
Input	0.41±0.14	0.85±0.53	1.71±0.24	1.13±0.30	0.00	0.00
CycleGAN [6]	0.42±0.12	0.43±0.14	1.01±0.23	1.11+0.23	1.12±0.20	1.33±0.37
FastCUT [17]	0.61±0.22	0.81±0.21	1.19±0.27	1.11±0.25	2.00±0.58	2.34±0.55
Colores et al. [5]	0.31±0.08	0.40±0.12	1.27±0.27	1.19±0.26	**2.57**±**0.55**	**3.09**±**0.56**
Cycle-Dehaze [7]	**0.28**±**0.20**	**0.29**±**0.08**	**2.10**±**0.30**	**2.11**±**0.22**	1.65±0.42	1.77±0.55
DeSmoke-LAP (IC)	0.42±0.15	0.41±0.17	0.97±0.20	1.00±0.27	1.02±0.15	1.37±0.33
DeSmoke-LAP (DC)	0.41±0.15	0.41±0.15	0.99±0.25	1.11±0.24	1.06±0.12	1.38±0.31
DeSmoke-LAP (IC+DC)	**0.41**±**0.14**	**0.41**±**0.14**	**1.10**±**0.20**	**1.13**±**0.26**	**1.09**±**0.20**	**1.41**±**0.30**

**Table 2:** Comparative analysis using the video clips’ dataset, reporting mean and standard deviation of the 3 metrics

	FADE	JNBM	REA
**Input**	0.95±0.50	2.80±1.09	0.00
CycleGAN [6]	0.40±0.2	1.05±0.15	1.37±0.35
FastCUT [17]	0.59±0.27	1.11±0.20	1.13±0.18
Colores et al. [5]	0.36±0.09	1.18±0.20	**5.60**±**1.75**
Cycle-Dehaze [7]	**0.28**±**0.05**	**2.03**±**0.19**	1.60±0.34
DeSmoke-LAP (IC)	0.38±0.77	1.00±0.19	1.70±1.17
DeSmoke-LAP (DC)	0.37±0.83	1.07±0.18	1.74±1.00
DeSmoke-LAP (IC+DC)	**0.36**±**0.79**	**1.08**±**0.15**	**1.79**±**1.23**

Lower FADE, and higher JNBM and REA values are better

We also perform quantitative testing on video clips extracted from each video, reported in supplementary material Sec. 3, and average results over all folds and clips are presented in Table 2. Colores et al. [5] achieved overall best performance by referenceless metrics, whereas the proposed method outputs fine results compared to other approaches. Our method, Cycle-Dehaze method and Colores’s method perform well in both clear and hazy classes on FADE value. Delving into JNBM and REA metrics, our method falls behind Colores’s method because of low sharpness and poor performance of edge restoration. Since referenceless metrics are designed from natural images which are largely different from surgical images, these may not be true indicative of surgical images quality.

**Fig. 5:**
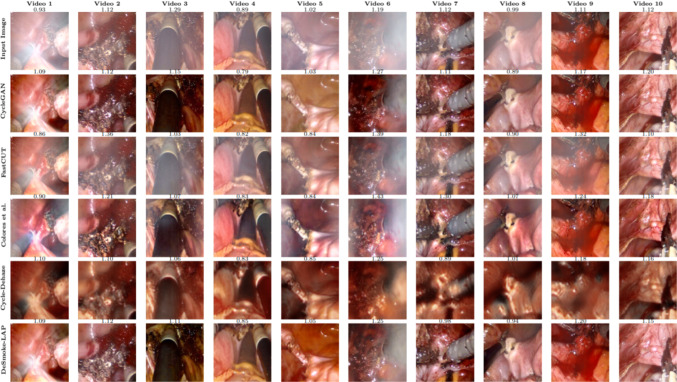
Qualitative comparison of the DeSmoke-LAP with the existing approaches using representative frames from 10 video clips where JNBM value of each image is also displayed

We further investigated video clips for qualitative comparison (Fig. 5). The video results from these clips are available on the provided link 1 and in the supplementary video. We divided testing images into three main groups based on their density of smoke, which includes light, medium and heavy. Three samples were picked from each group for visual contrast (as shown in Supplementary Sec. 4), annotated with JNBM value. We observe the density of smoke in the recovered image and colour variation between the input and output. We also considered the reliability and harmony of the recovered image, meaning the synthetic data must look like real data.

**Fig. 6:**
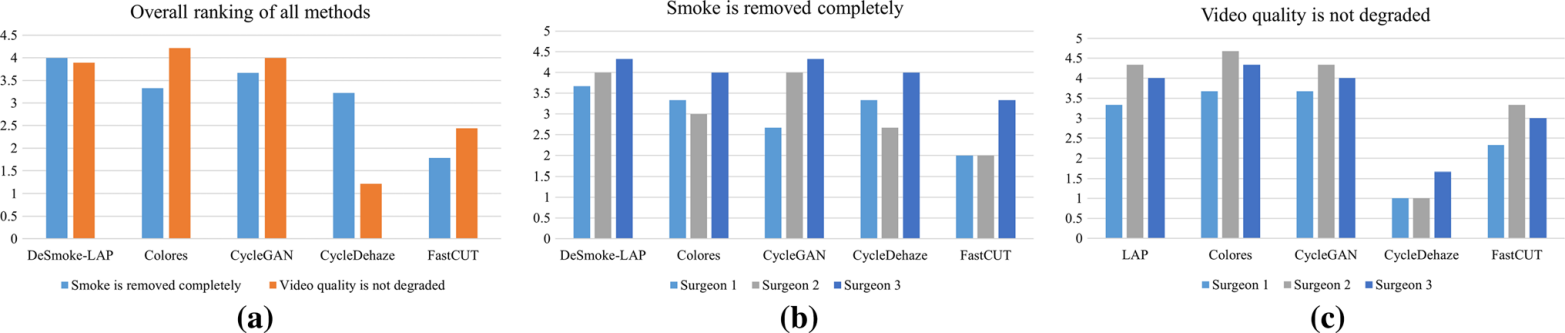
Qualitative analysis performed through the user study where the participants (surgeons) rated the output videos from each method based on two statements, **a** overall ranking of all methods under comparison, **b** individual surgeon’s ranking on statement 1: smoke is removed completely, **c** individual surgeon’s ranking on statement 2: video quality is not degraded

All methods showed positive effects on desmoking in surgical images, except FastCUT [17] failed to completely eliminate the smoke from hazy images (Fig. 5 and supplementary video$^1$). Cycle-Dehaze [7] works well on dehazing but produced low-quality outputs. The proposed DeSmoke-LAP visually outperformed Colores et al. [5] and took the lead in optimisation of dark pixels where most smoke was detected and removed (see suppl. video). In terms of JNBM, Colores et al. appeared to be better or comparable with the DeSmoke-LAP because sometimes it generates sharper images clipped at lower intensities, leading to information loss due to visually attenuated desmoked images. Referenceless metrics fail in evaluating these details which are aesthetically not appealing during visualisation.

A user study is included to access the acceptance of the proposed method by direct end-users, i.e. surgeons who routinely perform laparoscopic surgeries. The user study focuses on two statements related to the performance of smoke removal and the video quality after the process. An online video questionnaire was set up in which we showed the original and output video from each method side-by-side and asked the participants to rate the output video ‘statement 1: smoke is removed completely’ and ‘statement 2: video quality is not degraded’. Three surgeons participated in this study and showed their agreements to statements by ranking from 1 to 5, where 1 indicates strongly disagree and 5 indicates strongly agree. The average score achieved by each method on the two statements is reported in Fig. 6. From this figure, we observe that our proposed method slightly outperformed (score: 4) others in statement 1, followed by cycleGAN (score: 3.67) and Colores et al. (3.33). This suggests the participants visibly noticed the removal of smoke from DeSmoke-LAP. Some residue smoke remained on the corner of the output videos by Colores et al., but this was overlooked by the participants. Since video quality is not noticeably degraded in ours, Colores et al. and cycleGAN, all these three methods received comparable rankings for statement 2. FastCUT and Cycle-Dehaze were the worst in both statements. We further analyse the agreement of each surgeon in ranking the five methods under comparison using the two statements. The results are shown in Fig. 6b and c. This figure shows that all three surgeon participants rankings are comparable for all five methods and are in agreement with each other. These qualitative results obtained directly from the end-users (surgeons) suggest that our approach is acceptable and successful in removing smoke from laparoscopic videos while maintaining the original quality of the video. The superior response for statement 1 also justifies the feasibility of the two loss functions in the adversarial network that we specifically added to model smoke and remove any residual smoke.

The proposed DeSmoke-LAP showed its strength in removing partial smoke on the image. Two loss functions designed for desmoking in surgery domain can also be implemented on other frameworks, for example FastCut, without major alterations in the method itself. Experiments show that IC and DC have larger effect even under non-uniform lighting in surgery data and aid to remove smoke on dark components. Referenceless metrics fail to describe detailed desmoked information of the image but only summaries the quality, thus we have to rely on the visual evaluation, whereas quantitative results act as the assistance. When looking at Video 6 in Fig. 5, it shows that results by CycleGAN and proposed method received comparative high value as that by Cycle-Dehaze when much smoke remained on the frame, suggesting that presence of unremoved smoke artificially improve referenceless metrics. When Colores et al.’s method produced excellent quantitative outputs, only some parts of smoke are removed perfectly. Outputs by Cycle-Dehaze failed to achieve high-quality and vivid vision needed for laparoscopic surgery. DeSmoke-LAP guarantees the smoke is processed in accordance with its blur level, and the coordination of the colour is not be affected dramatically. Future work involves retaining the original resolution of the laparoscopic video to obtain high-quality desmoked images that would be beneficial for clinical use and use of larger dataset for further improving the method’s robustness.

## Conclusion

Compared to traditional open surgery, laparoscopic robot-assisted surgery manages the operation through tiny incisions by robot arms, finding a wide application in medicine. However, smoke generated due to electrocauterisation during laparoscopic surgery has been a potential risk to patients and surgeons. To address this issue, we proposed DeSmoke-LAP, a method for virtually removing smoke in laparoscopic surgery for enhancing intraoperative imaging. DeSmoke-LAP performed unpaired image-to-image translation between hazy and clear images based on cycle-consistency generative adversarial network. Unlike existing image dehazing methods, DeSmoke-LAP does not rely on synthetic paired data and atmospheric scattering model. Instead, we introduced two additional losses in the discriminator that assist to estimate the remaining smoke in the generated image by inter-channel discrepancies and dark channel prior. We quantitatively and qualitatively compared DeSmoke-LAP with the state-of-the-art image methods through five-fold cross-validation. Referenceless metrics have been introduced to evaluate the generated data in surgery domain; however, these metrics evaluate general image quality, but fail to evaluate the smoke status, which is the main task of this paper. Thus, we should rely on the visual evaluation since the foremost target is smoke removal, whereas the quality performance is the secondary consideration. The trained models were also tested on video clips, and we observed that desmoked frames by DeSmoke-LAP appeared to be consistent and smooth throughout the video, outperforming other methods. According to the user study performed to validate the level of smoke removal and the video quality, we found that participants (surgeons) were satisfied with the achievement by the proposed method. The participants show strong agreement for our proposed method, forecasting that it can be easily accepted by surgeons and clinicians to support laparoscopic surgeries. DeSmoke-LAP generated better quality, colour and contrast outputs without any clipping or attenuation, leading to visually meaningful desmoked results. The dataset and code will be made publicly available, providing a benchmark for desmoking in laparoscopic surgery.

## Footnotes

1 Code and visual comparison on video clips: https://www.ucl.ac.uk/interventional-surgical-sciences/weiss-open-research/weiss-open-data-server/desmoke-lap.

## Declarations

### Code availability

Code to be released with this paper.
